# Canadian Biomass Burning Aerosol Properties Modification during a Long-Ranged Event on August 2018

**DOI:** 10.3390/s20185442

**Published:** 2020-09-22

**Authors:** Christina-Anna Papanikolaou, Elina Giannakaki, Alexandros Papayannis, Maria Mylonaki, Ourania Soupiona

**Affiliations:** 1 Laser Remote Sensing Unit, Physics Department, School of Applied Mathematics and Physical Sciences, National Technical University of Athens, 157 80 Zografou, Greece; apdlidar@central.ntua.gr (A.P.); mylonaki.mari@gmail.com (M.M.); soupiona.rania@gmail.com (O.S.); 2 Department of Environmental Physics and Meteorology, Faculty of Physics, National and Kapodistrian University of Athens, 157-72 Zografou, Greece; elina@phys.uoa.gr; 3 Finnish Meteorological Institute, P.O Box 1627, 70211 Kuopio, Finland

**Keywords:** CALIPSO, Lidar, biomass burning aerosol, Canadian smoke, Canadian wildfires 2018

## Abstract

The aim of this paper is to study the spatio-temporal evolution of a long-lasting Canadian biomass burning event that affected Europe in August 2018. The event produced biomass burning aerosol layers which were observed during their transport from Canada to Europe from the 16 to the 26 August 2018 using active remote sensing data from the space-borne system Cloud-Aerosol Lidar and Infrared Pathfinder Satellite Observation (CALIPSO). The total number of aerosol layers detected was 745 of which 42% were identified as pure biomass burning. The remaining 58% were attributed to smoke mixed with: polluted dust (34%), clean continental (10%), polluted continental (5%), desert dust (6%) or marine aerosols (3%). In this study, smoke layers, pure and mixed ones, were observed by the CALIPSO satellite from 0.8 and up to 9.6 km height above mean sea level (amsl.). The mean altitude of these layers was found between 2.1 and 5.2 km amsl. The Ångström exponent, relevant to the aerosol backscatter coefficient (532/1064 nm), ranged between 0.9 and 1.5, indicating aerosols of different sizes. The mean linear particle depolarization ratio at 532 nm for pure biomass burning aerosols was found equal to 0.05 ± 0.04, indicating near spherical aerosols. We also observed that, in case of no aerosol mixing, the sphericity of pure smoke aerosols does not change during the air mass transportation (0.05–0.06). On the contrary, when the smoke is mixed with dessert dust the mean linear particle depolarization ratio may reach values up to 0.20 ± 0.04, especially close to the African continent (Region 4).

## 1. Introduction

Atmospheric aerosols are a fundamental part of the Earth’s atmosphere with a significant impact on climate, as they can interact with radiation and clouds [1,2,3]. Atmospheric aerosols are difficult to characterize due to their highly variable spatio-temporal distribution and their production mechanisms. Moreover, natural and anthropogenic aerosols from different sources can be mixed or aged during transport, which furthermore influences their properties [1,2,3].

Wildfires, agricultural burnings and increased use of wood as fuel for heating are the major sources of atmospheric aerosols related to biomass burning [4]. Biomass burning aerosols (BBs) can directly scatter and absorb solar radiation (so-called direct effect). They can also interact with clouds and change their properties, such as cloud albedo, precipitation efficiency and lifetime (so called indirect effect) [2,5,6,7].

Furthermore, increased attention is drawn to the fact that a lot of health risks of great importance are related with the increase of biomass burning. BBs can be produced by natural sources and/or anthropogenic processes. On a global scale, central and southern Africa, North America, Canada and Siberia (Russian Federation), the Midwest and the Amazon in Brazil, form the largest sources of biomass burning aerosols on Earth, mostly during the dry seasons [8,9,10].

It is well known that the kind of vegetation burning influences the released amount of soot (Black carbon: BC) and the size of the emitted smoke aerosols and, thus, their optical and physico-chemical properties [7,11]. BBs are one of the key aerosol types in climate research and due to the scarcity of relevant data in the literature, the vertical stratification of their optical, microphysical and chemical properties in free tropospheric layers is urgently needed [4].

Furthermore, smoke plumes from forest fires can be injected directly into the planetary boundary layer (PBL) and the free troposphere [12,13], or even to the lower stratosphere [14,15]. Long-range transport mechanisms, found in the free troposphere and lower stratosphere can distribute these smoke aerosols hemispherically [7,16,17].

This fact leads to an important issue regarding the transformation processes undertaken by the smoke aerosols, such as coagulation, condensation, and gas-to-particle conversion frequent during long-range transport leading to changes in their size and therefore to their optical properties [4].

In recent years, an increasing number of investigations focusing on the retrieval of the vertical profiling of the BBs’ geometrical and physico-chemical properties in the case of large fires all over the world is based on ground-based and spaceborne lidar systems [7,14,18,19,20,21,22,23,24]. In this paper, we investigate the modification of the vertical profiles of the BBs’ geometrical and optical properties during a long-range biomass burning event that occurred over Canada from the 16 to 26 August 2018, as observed by the Cloud-Aerosol Lidar and Infrared Pathfinder Satellite Observation (CALIPSO) satellite, following the BBs from their source along their pathway towards Europe. The mean values of smoke plumes’ altitude, and the relevant values of BBs’ aerosol backscatter coefficients (β), the particle linear depolarization ratio (LPDR) at 532 nm, as well as the backscatter-related Ångström exponent (AE 532/1064 nm) are presented and discussed during this intercontinental transport.

## 2. Methodology and Data

### 2.1. Smoke Event Description

In 2018, the Canadian region of British Columbia (BC) experienced its worst fire season on record since 2115 fires burned over 1.35 million hectares. This large fire event surpassed the 2017 fire season—previously the largest burned area—in which over 1.22 million ha were burned. The fires of 2018 in BC accounted for about 60% of the total burned area in Canada in 2018, compared to an average of 7% over the 1990 to 2018 period [25]. Smoke emitted from these fires largely contributed to the poor air quality measured in the province during the fire period of April to September. All air quality measuring stations had at least one day where the Air Quality Health Index (AQHI) reached 7 or even higher values. Furthermore, most of these stations preserved these high values for more than seven days, while three of them for almost a month (British Columbia’s forest fires, 2018). On the 16 August 2018 in BC 559 wildfires were active, leading the local BC. government to declare this province at the state of emergency. These smoke aerosol plumes travelled across the Atlantic Ocean and affected the western coasts of Europe on the 25 and 26 August.

In the frame of this paper, this intercontinental transport event will be studied with the synergy of satellite observations and models: data from the Cloud-Aerosol Lidar with Orthogonal Polarization (CALIOP) lidar system on board the CALIPSO satellite, the Moderate Resolution Imaging Spectroradiometer (MODIS), instrument on board the Terra and Aqua satellites, the HYSPLIT trajectory model and the NASA’s tool Giovanni will be used to study the source, the transportation and the modification of the geometrical and physical properties of the detected BBs.

### 2.2. Satellites, Models and Tools

**Figure 1:**
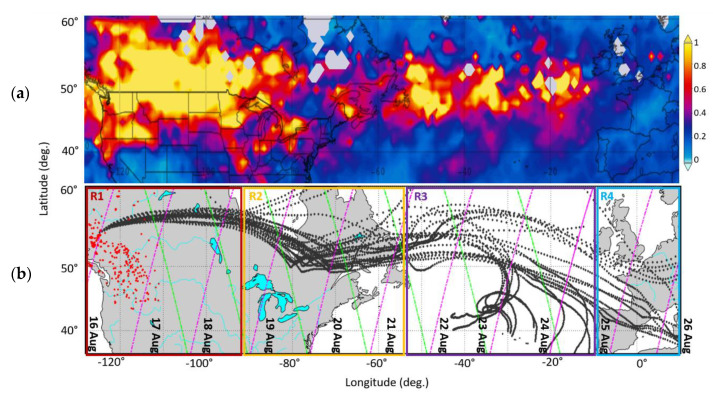
(**a**) Time Averaged Map of Combined Dark Target and Deep Blue AOD at 550 nm for land and ocean: Mean daily 1° [MODIS-Aqua MYD08_D3 v6.1] from 16 August 2018 and for a 10 day period. (**b**) The 10 day forward HYSPLIT trajectories starting on 16 August and ending on 10 August. The red dots correspond to active fires observed in BC, Canada, by MODIS with confidence greater than 80%. Magenta and green lines correspond to nighttime and daytime CALIPSO orbits, respectively. Red, yellow, purple and cyan boxes correspond to the four subregions (R1–R4) of the smoke motion that will be further analyzed in Section 3.2.

MODIS is flying on two satellites in orbits that complement each other and provides the diurnal variation of the rapidly varying atmospheric parameters for climate and global change studies with almost complete global coverage in one day [26]. In this study active fire data from MODIS Terra and Aqua, distributed through the Fire Information for Resource Management System (FIRMS), were used to analyze the distribution of fires in Canada during the studied period. In Figure 1b we present with a red dot the location where MODIS detected at least one fire event during the compositing time period, with confidence greater that 80% (The fire maps are available from NASA’s Earth Observing System Data and Information System (EOSDIS) (https://firms.modaps.eosdis.nasa.gov). The period covered by the fire map shown was set to 8 days, from 8 to 16 August 2018 coinciding with the period of intense wildfires in Canada.

Aerosol observations from several space-borne instruments can be accessed through the Giovanni tool, which is a data exploration system for visualization, and analysis of NASA Earth Science data (https://giovanni.gsfc.nasa.gov/giovanni/) [27]. The Giovanni tool was used to verify the path of smoke plume motion and visualize the AOD at 550 nm (based on MODIS (Aqua) data, with a temporal resolution of 24 h and spatial resolution of 1° (Figure 1a). In this figure we see that AOD values at 550 nm were greater than 0.825 close to the burning areas, while at the time smoke was reaching the European continent these values were still increased (>0.413).

**Figure 2:**
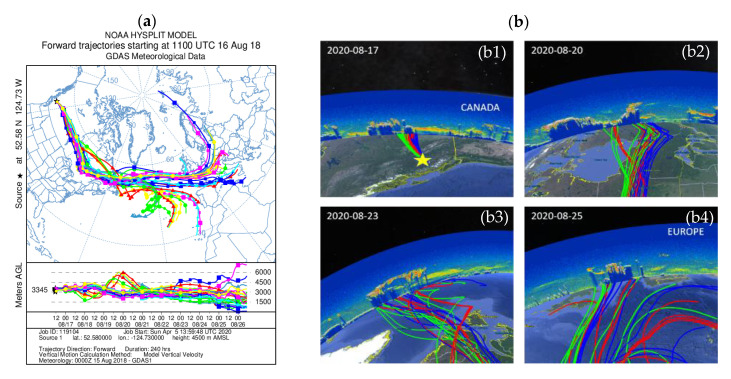
(**a**) 10-day forward ensemble air mass trajectories starting on 16 August (11:00 UTC), as provided by HYSPLIT. Different color-lines of the forward trajectories correspond to trajectories for all-possible offsets in longitude, latitude and altitude according to the ensemble analysis). (**b**) 10-day-forward ensemble air mass trajectories (foreground) over plotted along with selected CALIPSO curtains.

The HYSPLIT model “ensembles” has been an attractive approach to study an atmospheric transport (https://www.ready.noaa.gov). The “ensemble” method, that was used for the analysis of the forward air mass trajectories, is created by slightly offsetting the meteorological data to test the sensitivity of the advection calculation to the gradients in the meteorological data fields. Multiple trajectories start from the selected starting point, and each member of the trajectory ensemble is calculated by offsetting the meteorological data by a fixed grid factor. As a result, 27 members occur for all-possible offsets in longitude, latitude and altitude. This can work as an approximation of the true flow field [28,29]. One indicative forward air mass trajectory is shown in Figure 1 and Figure 2. The coordinates and altitudes of the smoke plumes were observed over Canada and categorized as smoke aerosols by the CALIPSO algorithm. These coordinates and altitudes were used as initial values for the model. The air mass starting point was placed at 52.6° N and 124.7° W, for this indicative trajectory, and the height was at 4500 amsl. The vertical motion calculation method used was the model vertical velocity along with the meteorological data GDAS1 (Global Data Analysis System). The duration of the trajectory was 240 h forward. The trajectory analysis showed the path that the smoke followed to reach Europe.

The CALIPSO satellite was the main tool used in this study, as it offers the unique opportunity of studying such a dynamic event, from its source region and across an intercontinental path passing over the Atlantic Ocean, where no ground-based lidars exist. CALISPO is a polar-orbiting satellite flying at an altitude of about 705 km, with a laser footprint covering 0.2% of the Earth’s surface during one full cycle. The CALIOP elastic backscatter lidar system, on board the CALIPSO satellite, emits simultaneously at 532 and 1064 nm (https://www-calipso.larc.nasa.gov/) is able to provide the vertical structure of aerosols along with their optical properties [30]. In this work, we used Level 2 CALIPSO products, specifically the Lidar Level 2 Vertical Feature Mask data product (VFM) and the Lidar Level 2 Aerosol Profile data products (APro) of version 3.40, which is provided in horizontal resolution of 5 km. The VFM product was the one used in order to separate the atmospheric aerosol layers, that the CALIPSO algorithm categorized as smoke, from the other types of aerosols. The mean altitude of the corresponding smoke layers was also obtained by the VFM product. Afterward, the optical properties used for the analysis were obtained by the Apro product. The nighttime and daytime orbits used in this study are presented with the magenta and green lines in Figure 1b.

### 2.3. Methods and Data Analysis

Since the active fires in the Canadian region were detected by MODIS, the CALIPSO orbits were used, at first, to observe the smoke layers in this region, near the wildfires area. The night and day orbits were used in order to track the transport of the smoke plume towards Europe, from 16 to 26 August. The plume was observed initially over the BC on the 16 August (11:00 UTC) between 45–56° N and 123–127° W, within the height region 2–5 km amsl. In Figure 2(b1–b4), we present the orbits of CALIPSO, along with the forward ensemble air mass trajectory.

The CALIPSO “curtains” shown in the background represent the vertical distribution of the total attenuated backscatter coefficient at 532 nm, while in the foreground, the forward air mass trajectories show the path that the smoke plume followed, from Canada to Europe in a 10-day period.

The observations of CALIOP were used to track the biomass burning plumes throughout their transport from Canada to Europe. In order to study the geometrical and optical properties of the smoke layers, the main region of smoke’s spatial distribution was limited from 38° N to 58° N and from 125° W to 10° E. The nighttime and daytime CALIPSO orbits where used to follow the smoke aerosol layers. The horizontal averaging applied to the CALIOP data was 105 km, in order to enhance the detection of the aerosol layers. For the data analysis, we used Atmospheric Volume Description (AVD) to screen out any other scatterer apart from aerosols. The standard cloud-aerosol discrimination (CAD) score, reported in the CALIPSO products range between −100 and 100. The sign of the CAD score indicates the feature type: positive values signify clouds, while negative values signify aerosols. The absolute value of the CAD score provides a confidence level for the classification(https://www.calipso.larc.nasa.gov/resources/calipso_users_guide/data_summaries/profile_data.php). To be on the safe side and exclude any cloud contamination, CAD score was set between −80 and −100.

**Figure 3:**
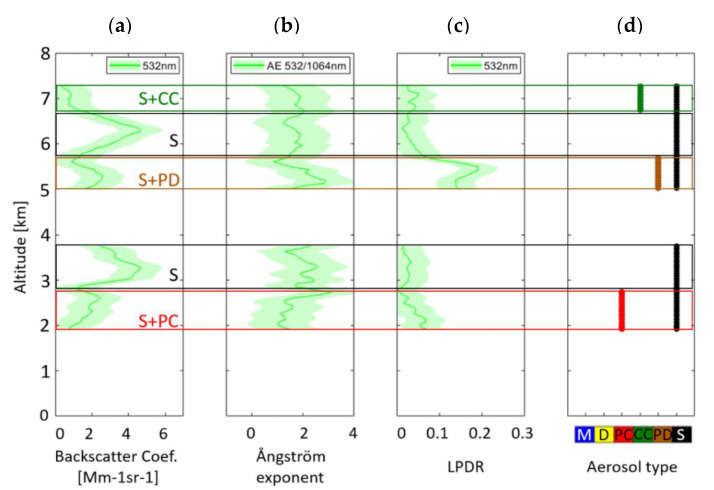
Vertically resolved aerosol optical properties: (**a**) β at 532 nm, (**b**) β-related AE 532/1064 nm, (**c**) LPDR at 532 nm and (**d**) aerosol typing according to the CALIPSO algorithm (M: marine, D: Dust, PC: Polluted Continental, CC: Clean Continental, PD: Polluted Dust, S: Smoke), as retrieved from the nighttime CALIOP orbit on 20 August. The coordinates used to derive these properties were 42.5° N and 73.8° W. Green shadowed lines correspond to the standard deviation for each aerosol property.

The vertically resolved particle optical properties (β and LPDR at 532 nm and AE 532/1064 nm) were retrieved by the CALIPSO nighttime and daytime orbits. The retrievals were made per 2^°^ latitude and longitude, along the CALIPSO orbits. The dates of the orbits used are presented in Figure 1b. Magenta and green lines correspond to nighttime and daytime CALIPSO orbits, respectively. The retrievals were made per 2° latitude and longitude, along each one of the CALIPSO orbits used. Profiles not containing any smoke layers were excluded. However, most of them, usually, included more than one smoke layer, in different heights. As mentioned before, the minimum spatial averaging of the CALIPSO product is 5 km and the average we used was 105 km. While the average can be applied in intensive aerosol parameters (β and LPDR at 532 nm and AE 532/1064 nm) it can’t be applied in the typing product. Thus, more than one aerosol type was attributed in each layer, as it can be seen in Figure 3d.

As an example of the aforementioned retrievals, the vertically resolved aerosol optical properties and the corresponding subtypes are presented in Figure 3 as derived from the nighttime CALIPSO orbit on 20 August 2018, 07:03, 42.5° N and 73.8° W. As it is shown in this figure, at least 5 aerosol layers were observed (Figure 3d). Two of them were categorized as pure smoke (S), while 3 as smoke mixed with clean continental aerosols (S+CC), smoke with polluted dust (S+PD) and smoke with polluted continental (S+PC).

Initially, all layers containing smoke, according to CALIPSO aerosol typing algorithm, were isolated and analyzed. However, as all the studied layers contained either pure smoke, or smoke mixed with other aerosols, 6 new categories were added in this work: pure smoke (1), smoke mixed with polluted dust (2), with desert dust (3), with clean continental (4), with polluted continental (5) and with marine aerosols (6). In that way, two aerosol categories containing smoke mixed with dust have emerged, the first containing smoke mixed with polluted dust and the second containing smoke mixed with dessert dust. In the first category the smoke is mixed with dust that is already mixed with smoke or other continental polluted aerosols, while the second category is a mixture of two pure aerosol types: smoke and dessert dust. Differences between these categories occurred, mostly, based on their LPDR and LR values.

The analysis applied on the data sets was the box plot. The box plot analysis is useful for analyzing data sets as large as those studied here, as they provide a visual summary of the data and an easy identification of the median and mean values, the dispersion of the data set, and signs of skewness. The minimum and maximum values are found at the end of the whiskers and are useful for providing a visual indicator regarding the range of the data. The 75th percentile and 25th percentile values indicate the values at which 75% of the data are above it and 25% below.

The box plot analysis was first applied on the whole dataset of the event, then on the four subregions that the smoke region was divided and finally, only on the pure smoke aerosol layers which were identified. Regarding the four subregions (R1 to R4), the first subregion (R1) contains all the smoke layers located over the active fires and up to two days eastward travel from the fire source. The second subregion (R2) is the one containing all the smoke layers eastward of the first region and up to the eastern Canadian coasts. The third subregion (R3) contains the smoke layers which cross the Atlantic Ocean and finally the fourth subregion (R4) contains the smoke layers that reached the western coasts of Europe (in Figure 1 the four subregions are presented with the corresponding colors).

## 3. Results and Discussion

### 3.1. Mixed Smoke Layers Analysis

**Figure 4:**
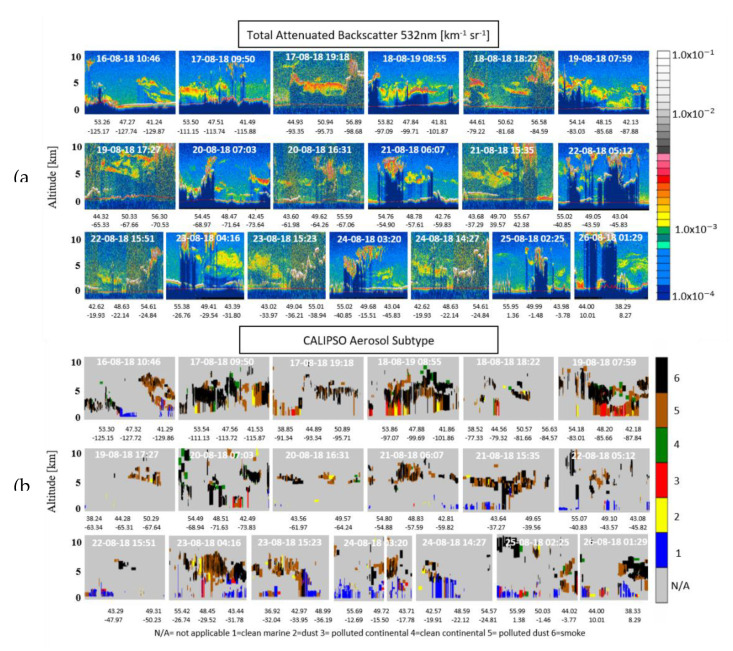
(**a**) CALIPSO total attenuated backscatter coefficient at 532 nm and (**b**) aerosol subtypes versus altitude, latitude and longitude for nighttime and daytime orbits (16–26 August 2018).

**Figure 5:**
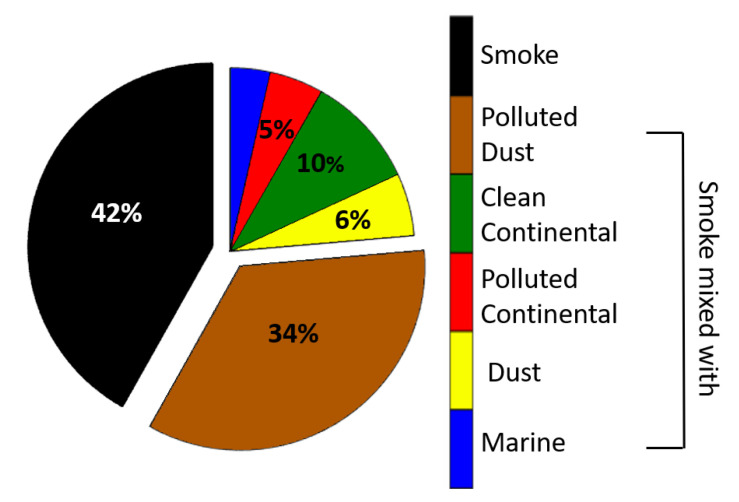
Percentages of aerosol layers mixing types for the total event, types found in percentages less than 3%, are not presented with numbers in the figure.

The total attenuated aerosol backscatter coefficient at 532 nm for each day of the tracked smoke plume, in the time period 16–26 August 2018, are presented in Figure 4, where the corresponding color bar on the right of the figure indicates the intensity of the aerosol load (e.g., the atmospheric aerosol layers are delineated by the yellow, orange and light-red colors). These aerosol layers were identified as smoke, pure or mixed with other aerosol types, by the CALIPSO algorithm. The corresponding aerosol types, according to CALIPSO’s algorithm, are presented in Figure 5. With black color are represented the smoke layers, with brown and yellow colors the polluted dust and desert dust, respectively, with green and red colors the clean and polluted continental and, finally, with blue color the marine layers.

The total number of the corresponding profiles obtained was 715. We then focused on the aerosol layers of pure smoke or mixtures of smoke aerosols as categorized by CALIPSO. In total, we found 745 layers of smoke: 312 of them were identified as pure smoke, 257 as smoke mixed with polluted dust, 74 as smoke mixed with clean continental aerosols, 35 as smoke mixed with polluted continental, 41 as smoke mixed with dessert dust and, finally, 26 as smoke mixed with marine aerosols, the percentages are extensively shown in Figure 5. 

**Figure 6:**
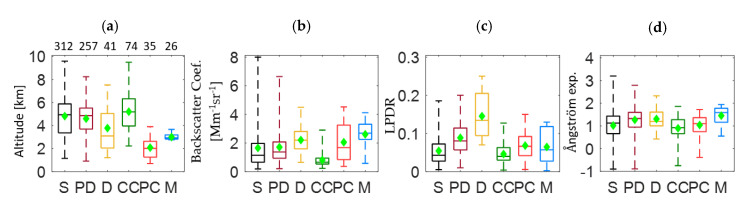
Aerosol layers mixtures according to (**a**) their altitude (amsl.), (**b**) β at 532 nm, (**c**) LPDR at 532 nm, (**d**) AE 532/1064 nm, related to β, for the total event (S: pure smoke layers, PD: smoke mixed with polluted dust layers, CC: smoke mixed with clean continental layers, PC: smoke mixed with polluted continental layers, D: smoke mixed with dust layers and M: smoke mixed with marine layers).

Furthermore, we proceeded with the box-plot analysis of the aerosol layers’ properties concerning their geometrical height properties (amsl.), the values of β and LPDR at 532 nm, as well as the β-related AΕ 532/1064 nm. This analysis was performed using all available biomass burning layers and their mixtures to obtain representative values of aerosol properties during a large-scale biomass burning event. The results are presented in Figure 6 containing the box plot analysis that represents the distribution of the values of the aerosol properties mentioned before for all aerosol layers studied within this event. Each color of the boxes corresponds to the aerosol type of pure smoke and different smoke mixtures. Green rhombus correspond to the mean values. The horizontal line inside the box represents the median values, while the smallest and largest values are put at the end of the whiskers. The box limits (up and down) correspond to the 75th percentile and 25th percentile values that indicate the values at which 75% of the data are above it and 25% below. The number of layers for all mixing types are presented above the altitude box.

From the analysis of Figure 6a, it was found that mean values of the layers’ altitude ranged between 2.1 ± 0.9 km (smoke mixed with polluted continental aerosols) and 5.2 ± 1.5 km (smoke mixed with clean continental aerosols). The 75% of the smoke mixed with polluted continental aerosol layers was found at altitudes greater than 2.6 km and 25% at altitudes lower than 1.3 km amsl. Specifically, the smoke layers containing polluted continental aerosols were probed at lower altitudes. Smoke mixed with clean continental aerosol layers were found at altitudes greater than 6.3 km in 75% and only 25% were found at altitudes lower than 4 km amsl. This could be explained by the fact that the sources of the polluted continental aerosols are, mostly, at near ground levels. Moreover, the pure smoke aerosol layers were found mostly above 5.9 km amsl. (75%).

Concerning the mixtures of smoke with marine aerosols the mean altitude of these layers was found at about 2.9 ± 0.3 km amsl. (2.8 to 3.7 km amsl.), which seems quite improbable, as typically, the marine boundary layer (MBL) does not exceed 1.5 km height [31,32]. Therefore, this could be due to a possible aerosol misclassification by the CALIPSO algorithm. In our case, layers of pure smoke, smoke mixed with polluted dust or with clean continental aerosols were observed at, approximately, the same altitude ranges, is in agreement with studies related to smoke injection height [13,16].

In general, the mean β values at 532 nm (Figure 6b) retrieved from each of the corresponding smoke layers, ranged from 0.8 to 2.6 Mm^−1^sr^−1^. On the other hand, layers including smoke aerosols mixed with dust, pure or polluted showed mean β values at 2.2 ± 0.9 and 1.7 ± 0.6 Mm^−1^sr^−1^, respectively. More precisely, for these smoke mixtures 75% of the β values were greater than 2.9 and 2.1 Mm^−1^sr^−1^. The β values of pure smoke layers appeared to be mainly (75%) greater than 2.0 Mm^−1^sr^-1^, with some values reaching even 8 Mm^−1^sr^−1^. The smoke mixed with clean continental aerosol layers showed the smallest β values (0.8 ± 0.5 Mm^−1^sr^−1^).

The mean values of the LPDR and AE (Figure 6c,d) indicate changes in the shape and size of the aerosols due to the transforming processes that smoke aerosols went through, during their long-range transport, as mentioned before. The LPDR values, that ranged between 0.04 to 0.15 and those of AE greater than 1 (not exceeding 1.9), are representative for smoke aerosols [33,34]. On the other hand, LPDR values, greater than 0.20 and AE values close to zero, indicate the presence of dust aerosols [1,19]. We further found that 75% of the pure smoke aerosols LPDR and AE values were greater than 0.07 and 1.4, respectively. The corresponding LPDR and AE values for the smoke aerosols mixed with polluted dust were 0.11 and 1.6, respectively. For the layers containing smoke mixed with clean and polluted continentals the LPDR and AE values were 0.06, 0.09 and 1.3, 1.4, respectively. In Table 1 are presented extensively all values obtained by the analysis of the data in Figure 6. An Appendix A is also found at the end of a paper containing all the information of the box plot analysis from the following sections as well (Table A1, Table A2, Table A3, Table A4 and Table A5).

In conclusion, the layers of smoke mixed with clean continental aerosol differentiated compared to the other aerosol categories, regarding the low value of β. This fact might explain the reason why CALIPSO algorithm classified these layers as clean continental ones. Furthermore, the smoke layers mixed with desert dust were those that seem to pole apart from the other types. The mean altitude of these layers was 3.9 ± 1.8 km amsl., and the AE 532/1064 nm mean value was similar to that of the other types, although it presented the smallest variation. However, the LPDR values of this category were the only ones which were greater than 0.20. Compared to the smoke mixed with polluted dust category, aerosol layers of smoke mixed with dessert dust were found in lower altitudes and were more depolarized (LPDR equal to 0.15 ± 0.06), but with the same AE mean values of the order of 1.3 ± 0.4.

### 3.2. Smoke Transportation Analysis per Region

**Figure 7:**
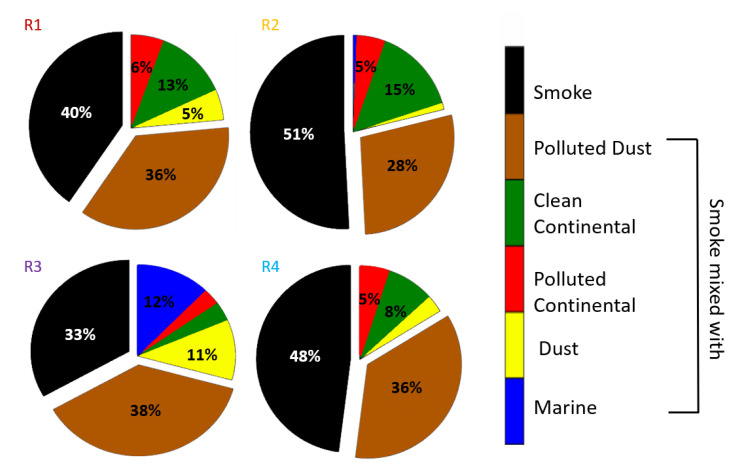
Percentages of the aerosol layers mixing types for the subregions (**R1**–**R4**). Aerosol types found in percentages less than 3%, are not presented with numbers in the figure.

For each one of these four subregions that the study region was divided, the studied layers were also analyzed to percentages per mixing type and per region. In R1, 263 aerosol layers were detected, from them 40% of which were identified as pure smoke and the rest 60% as smoke mixed with polluted dust, clean and polluted continental and dust. The R2 contained a 51% of pure smoke layers. In R3, 33% was categorized as pure smoke layers, 38% as smoke mixed with polluted dust and 12% as smoke mixed with aerosols. Finally, in R4, 48% of the layers were categorized as pure smoke and 36% as smoke mixed with polluted dust. The exact percentages are shown in Figure 7.

As discussed previously, the majority of the identified smoke layers were either pure or mixed with polluted dust. However, we have to note that the percentages of pure smoke layers are larger in the subregions R2 and R4 compared to R1, which is the active fire area. This could be explained by the fact that the first CALIPSO trajectory may have passed over the fires but did not pass at the exact time or place that the smoke layers were more intense. It could also be related to smoke layers from the same event or even layers originating from other fires, which may have contributed to already existing smoke layers in subregion R2. The thriving percentages of smoke mixed with polluted dust, in subregions R1 to R4, is something worth to be mentioned. This could be related to the mechanisms which are responsible for the mixing of dust with smoke during BB events. It has been shown that flaming fires may be efficient enough to mobilize the surface soil dust [35] and so dust can be elevated and thus mixed with the smoke. This could also lead to the conclusion that some of the layers were misclassified by the automated CALIPSO classification [36].

In contrast to the smoke layers mixed with polluted dust, smoke layers mixed with marine aerosols are observed only in subregions R2 and R3 in almost insignificant percentages (26 layers in total). This is quite expected and related to the injection of the marine aerosols into the lowermost part of the atmosphere, with a maximum of MBL height up to 1.5 km height [31,32] thus not mixing with smoke aerosols present, mostly, in the free troposphere. The percentages of clean and polluted continental aerosols mixed with smoke were found between 5–15% in each subregion, indicating insignificant contribution of the aerosol types to the smoke layers, as they were transported towards Europe.

**Figure 8:**
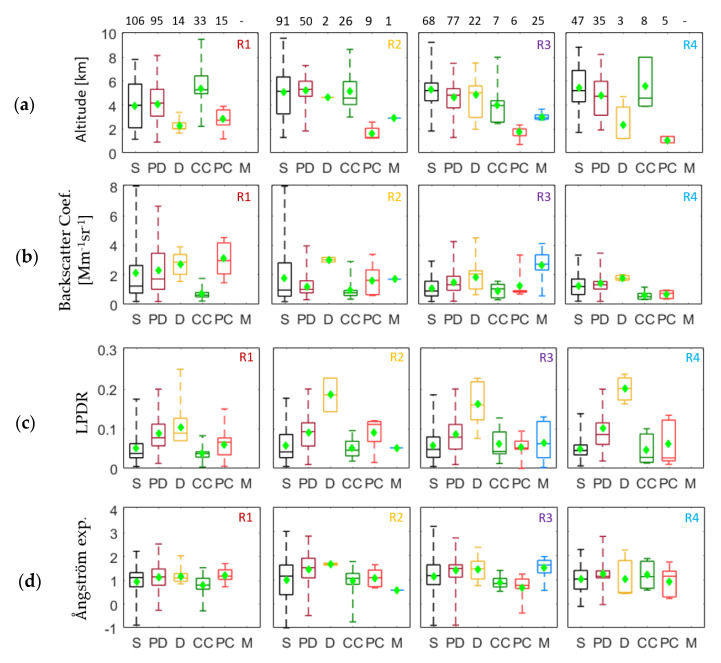
Aerosol layers mixtures according to (**a**) their altitude (amsl.), (**b**) β at 532 nm, (**c**) LPDR at 532 nm, (**d**) AΕ related to β (532/1064 nm). R1–R4 (left to right) correspond to the four subregions (S: pure smoke layers, PD: smoke mixed with polluted dust layers, CC: smoke mixed with clean continental layers, PC: smoke mixed with polluted continental layers, D: smoke mixed with dust layers and M: smoke mixed with marine layers).

In Figure 8 we present the box-plot analysis for the four subregions over which smoke was observed. Each colored box corresponds to the aerosol type of pure smoke and smoke mixtures. The mean, median, min, max values and 75th, 25th percentiles for all variables (altitude, β, LPDR and AΕ) are presented as mentioned before. The number of layers for all mixing types are presented above the altitude boxes within each subregion (R1 to R4: left to right).

Thus, in the R1 region (Figure 8) all aerosol subtypes (smoke and smoke mixtures) were found, except the marine aerosol mixtures. We found 106 pure smoke layers, 95 smoke mixtures with polluted dust, 33 mixed with clean continental, 15 mixed with polluted continental and 14 mixed with desert dust. The smoke layers and those mixed with polluted dust were found at mean altitudes of 4.0 ± 1.9 km and 4.1 ± 1.4 km, respectively. The clean continental layers were observed at mean altitude of 5.4 ± 1.5 km and the layers containing polluted continental and dust aerosols were found at 2.8 ± 0.9 km and 2.3 ± 0.5 km, respectively. The corresponding aerosol β mean values for all subtypes ranged between 0.7–3.1 Mm^−1^sr^−1^, while the mean LPDRs ranged from 0.04 ± 0.02 (for smoke with clean continental aerosols) to 0.10 ± 0.05 (for smoke mixed with dessert dust). Finally, the mean AΕ values ranged from 0.9 to 1.2.

In the R2 region all aerosol subtypes of smoke and smoke mixtures were observed. We found 91 pure smoke layers, 50 polluted dust smoke mixtures, 26 clean continental smoke mixtures, nine polluted continental smoke mixtures, two layers of smoke mixed with dust and one with marine aerosols. Smoke and polluted dust were detected at 5.1 ± 2.2 and 5.2 ± 1.1 km altitude, respectively, while smoke mixed with clean continental aerosols at 5.2 ± 1.5 km. Layers of smoke mixed with dust had a mean altitude of 4.7 ± 0.1 km, while smoke mixed with polluted continental and marine aerosols were found at 1.6 ± 0.6 and 2.9 km, respectively. The corresponding values of β ranged from 1.0 to 3.0 Mm^−1^sr^−1^. The LPDR values ranged from 0.05 to 0.18, while the AΕ mean values were found equal to 0.6 for smoke mixed with marine aerosol layers and 1.6 ± 0.1 for smoke mixed with dust aerosols.

In the R3 region all aerosol subtypes of smoke and smoke mixtures were, also, observed. We found 68 pure smoke layers, 77 polluted dust smoke mixtures, 22 layers of smoke mixed with desert dust, seven clean continental smoke mixtures, six polluted continental smoke mixtures and 25 layers of smoke mixed with marine aerosols. Pure smoke layers and those mixed with polluted and dessert dust were detected at 5.3 ± 1.5, 4.7 ± 1.2 and 4.9 ± 1.6 km, respectively. Clean continental layers were found at 4.0 ± 1.9 km, while the layers containing polluted continental and marine smoke mixtures were found at 1.8 ± 0.6 and 3.0 ± 0.3 km, respectively. The values of β ranged from 0.9 to 2.6 Mm^−1^sr^−1^, while the LPDR means ranged from 0.05 to 0.16. The AΕ mean values ranged from 0.7 to 1.5.

In the R4 region, over western Europe, all aerosol subtypes (smoke and smoke mixtures) were found except the marine aerosol mixtures. We found 47 pure smoke layers, 35 polluted dust smoke mixtures, eight clean continental smoke mixtures, five polluted continental and three smoke layers mixed with dessert dust. Smoke and polluted dust mixed layers were detected at 5.5 ± 2.0 and 4.8 ± 1.8 km altitude, respectively. The smoke mixed with clean continental aerosol layers and polluted continental were found at 5.6 ± 2.0 and 1.1 ± 0.3 km, respectively, while the desert dust smoke mixtures were detected at 2.4 ± 2.2 km. The corresponding β values ranged from 0.7 to 1.8 Mm^−1^sr^−1^. The mean LPDR values ranged from 0.05 to 0.20 (for pure smoke and smoke mixed with dessert dust, respectively), while those of AΕ ranged from 0.9 to 1.2.

**Figure 9:**
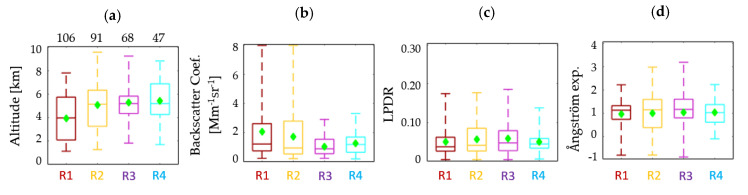
Pure smoke aerosol layers according to (**a**) their altitude (amsl.) and relevant optical properties: (**b**) β at 532 nm, (**c**) LPDR at 532 nm, (**d**) AΕ related to β (532/1064 nm). R1–R4 correspond to the four subregions.

According to Figure 6a and Figure 8a, we observe a large variability in the layers’ height. This could be explained by the fact that the BB injection heights can differ according to the itensity of the BB event. Studies based on CALIPSO data obtained over the mid and high latitudes, showed that BB plumes can be equally injected within the mixing layer (50%) and the free troposphere (50%) [13,16]. On the other hand, the LPDR values (Figure 9c) for the smoke mixtures with dust ranged from 0.10 ± 0.05 to 0.20 ± 0.04, in all subregions, which is in accordance with values previously found in the literature [19,21,37,38,39]. The relevant values for the pure smoke aerosols were found equal to 0.05 ± 0.04, again in agreement with literature findings [7,17,40]. As for the polluted dust smoke mixtures, the mean LPDR value was quite stable and equal to 0.09 ± 0.05, in all subregions. The rest of the smoke mixtures showed LPDR values (in all subregions) ranging from 0.04 ± 0.02 to 0.09 ± 0.04.

In general, the β-related AE (532/1064 nm) values regarding the biomass burning aerosols from different sources, pure or mixed, presented a large variability (from 0.8 to 2.2). The AΕ mean values obtained in this paper ranged from 0.8 to 1.2 for smoke mixed with other types of aerosols in R1. In R2, the AΕ mean values were found equal to 0.6 for smoke mixed with marine aerosol layers, and to 1.6 ± 0.1 for smoke mixed with dust aerosols. In R3 and R4 subregions, the values of AΕ ranged from 0.7 to 1.5 and 0.9 to 1.2, respectively, again in agreement with values found in the literature for pure smoke and smoke mixtures [22,38,40,41].

### 3.3. Pure Smoke Layers Properties

In the following section we will focus on the study of the modification of the pure smoke aerosol layers and the relevant optical properties during their travel from Canada to Europe. We found 312 pure smoke layers of which the mean altitudes are presented in Figure 9a. The relevant optical properties of pure smoke aerosols (β and LPDR at 532 nm, and the β-related AΕ (532/1064 nm)), are also presented in Figure 9b–d, respectively for each subregion. The four colors of the boxplots correspond to the four subregions (R1 to R4). Each colored box corresponds to the aerosol type of pure smoke and smoke mixtures. The mean, median, min, max values and 75th, 25th percentiles for all variables (altitude, β, LPDR and AΕ) are presented as mentioned before. The number of the pure smoke layers for each subregion is presented above the altitude box.

According to Figure 9, the aerosol layers identified as pure smoke were found to be 106 in R1, 91 in R2, 68 in R3 and 47 in R4 subregions. As expected, the number of pure smoke layers during the air mass transport from R1 to R4 diminishes, as we move away from the BB area. We also observe that the smoke layers’ height is increasing during its motion towards Europe, with mean values starting at 4.0 ± 1.9 km and reaching 5.5 ± 2.0 km height amsl. On the other hand, the mean value of β at 532 nm, is decreasing as expected from 2.1 to 1.1 Mm^−1^sr^−1^, while the LPDR and AΕ mean values, seem to keep a steady value in all subregions, around 0.05 ± 0.04 and 1.0 ± 0.6, respectively.

The optical properties of the pure smoke layers in all regions seem to agree well with values found in the literature for Canadian and North American (tropospheric) biomass burning events (Table 2). The mean LPDR value of 0.05 ± 0.04 is within the limits originated by literature values, indicating LPDR values lower than 0.05 [42] that can reach up to 0.14 [5]. According to Gross et al. (2015) [1], the LPDR at 532 nm for the Canadian Biomass Burning measurements was found at 7 ± 2%. Ancellet et al. [17] showed values that ranged from 0.02 to 0.08, while Ortiz-Amezcua et al. [7] presented values ranging from 0.05 to 0.10. The mean AE value obtained by this study is among the lowest found in the literature (1.0 ± 0.6), regarding tropospheric Canadian and North American BB events [41,42,43,44].

## 4. Conclusions

In this paper a long-range transport event of biomass burning aerosols was studied, where aerosol layers of pure biomass burning, and mixed smoke aerosols were detected and analyzed in a region spanning from the wildfire sources up to the European continent. Forward trajectory analysis and satellite fire observations were the main tools used in order to analyze the evolution of this biomass burning event. The CALIOP lidar on board the CALIPSO satellite was used to track the transport of the smoke layers. The altitude of the observed layers, the values β and LPDR at 532 nm, as well as those of the β-related AΕ (532/1064 nm) were fully studied.

From the 745 aerosol layers detected, 42% of them were identified as pure biomass burning aerosols. The remaining 58% were attributed to smoke mixed with: polluted dust (34%), clean continental (10%), polluted continental (5%), Saharan dust (6%) or marine aerosols (3%). The smoke layers observed by the CALIPSO satellite were found within a wide range of altitudes from 0.8 km up to 10 km height. Most of the layers’ altitude was found between 2.1 and 5.2 km amsl. The mean value of β at 532 nm, for every smoke mixing type ranged from 0.8 to 2.6 Mm^−1^sr^−1^, while the mean value of LPDR at 532 nm, ranged from 0.04 ± 0.02 (smoke mixed with clean continental aerosols in R1), indicating nearly spherical aerosols, to 0.20 ± 0.04 (for smoke mixed with desert dust in R4). The mean LPDR value at 532 nm concerning pure biomass burning aerosols was found equal to 0.05 ± 0.04. The mean value of the β-related AΕ (532/1064 nm) ranged for all smoke mixed layers between 0.8 to 1.6, while for pure biomass burning aerosols stayed constant at 1.0 ± 0.6, within each subregion.

The majority of the identified smoke layers were either pure or mixed with polluted dust. However, for the smoke mixed with polluted dust layers we found mean values of LPDR and AE, equal to 0.09 ± 0.05 and 1.3 ± 0.6, respectively, in all subregions. These values do not seem to be indicative of dust aerosols (even polluted), thus this kind of layering could be possibly misclassified by the CALIPSO algorithm. The percentages of the clean and polluted continental aerosols were found between 5–15%, in each subregion, with no significant contribution in the aerosol optical properties. We, also, found that the smoke layers mixed with desert dust was the aerosol type showing the most observable changes, mostly in the LPDR values. Another result of our study was that the marine aerosols were not found to be significantly mixed with smoke aerosols as they were, mostly, confined within the MBL. The altitudes where the marine smoke mixtures were found (3.0 ± 0.2 km), along with the values of AE (0.6 to 1.9), could also lead to a possible aerosol misclassification by the CALISPO algorithm. Additionally, we found that the shape and the size (as they result from the LPDR and AE values) of pure smoke aerosols are not significantly changing during this smoke aerosol transportation. Finally, the mixing of smoke with other aerosol types played the major role for the changes observed in the aerosol optical properties.

## Figures and Tables

**Table 1:** Descriptive statistics of the altitude, β, LPDR and AE values for different aerosol types for the total event: mean, standard deviation (std), median, maximum value (max), minimum value (min), 75th and 25th percentile for all variables.

	Type	Parameter	Mean	Std	Median	Max	Min	75th perc	25th perc
**Pure**	**S**	Alt [km]	4.8	2.0	4.9	9.6	1.1	5.9	3.4
		β [Μm^−1^sr^−1^]	1.7	1.6	1.1	8.0	0.2	2.0	0.7
		LPDR	0.05	0.04	0.05	0.19	0.01	0.07	0.03
		AE	1.1	0.7	1.1	3.2	−1.0	1.4	0.7
**Smoke mixed with**	**PD**	Alt [km]	4.6	1.4	4.9	8.2	0.9	5.5	3.7
		β [Μm^−1^sr^−1^]	1.7	0.6	1.4	6.6	0.2	2.1	0.9
		LPDR	0.09	0.05	0.08	0.20	0.01	0.11	0.06
		AE	1.3	0.6	1.3	2.8	−0.9	1.6	0.9
	**D**	Alt [km]	3.9	1.8	3.1	7.5	1.2	5.0	2.0
		β [Μm^−1^sr^−1^]	2.2	0.9	2.2	4.5	0.6	2.9	1.6
		LPDR	0.15	0.06	0.13	0.25	0.07	0.21	0.09
		AE	1.3	0.4	1.2	2.3	0.4	1.6	1.0
	**CC**	Alt [km]	5.2	1.6	5.2	9.5	2.2	6.3	4.0
		β [Μm^−1^sr^−1^]	0.8	0.5	0.7	2.9	0.2	0.9	0.5
		LPDR	0.05	0.03	0.04	0.13	0.01	0.06	0.03
		AE	0.9	0.5	0.9	1.9	−0.8	1.3	0.7
	**CP**	Alt [km]	2.1	1.0	2.0	3.9	0.7	2.6	1.3
		β [Μm^−1^sr^−1^]	2.1	1.4	1.7	4.5	0.4	3.2	0.8
		LPDR	0.07	0.04	0.07	0.15	0.01	0.09	0.03
		AE	1.0	0.4	1.7	1.7	−0.4	1.4	0.7
	**M**	Alt [km]	3.0	0.2	2.9	3.7	2.8	3.2	2.8
		β [Μm^−1^sr^−1^]	2.6	1.0	2.7	4.1	0.6	3.3	2.2
		LPDR	0.06	0.04	0.13	0.13	0.01	0.12	0.03
		AE	1.5	0.4	1.6	1.9	0.6	1.8	1.2

**Table 2:** Optical properties of smoke aerosols measured during Canadian and North American biomass burning events, as cited in the relevant literature (2002–today).

Reference	LPDR (532 nm)	AE (532/1064 nm)
[18] Wandiger et. al. (2002)	0.06–0.11	-
[43] Dubovic et al. (2002)	-	1.0–2.3
[41] Müller et al. (2005)	-	0–2.1
[42] Müller et al. (2007)	≤5%	1.0
[5] McKendry et al. (2011)	0.07–0.14	-
[44] Preißler et al. (2013)	-	2.2
[1] Groß et al. (2015)	7 ± 2%.	-
[44] Veselovskii et al. (2015)	-	-
[17] Ancellet et al. (2016)	0.02–0.08	-
[7] Ortiz-Amezcua et al. (2017)	5–10%	-
[18] Vaughan et al. (2018)	≤6%	-
This study	0.05 ± 0.04	1.0 ± 0.6

## Appendix A

**Table A1:** Descriptive Statistics for different aerosol types for Region 1 (R1): mean, standard deviation (std), median, maximum value (max), minimum value (min), 75th and 25th percentile for all variables.

Region 1									
	Type	Parameter	Mean	Std	Median	Max	Min	75th perc	25th perc
**Pure**	**S**	Alt [km]	4.0	1.9	4.0	7.8	1.1	5.8	2.1
		β [Μm^−1^sr^−1^]	2.1	2.0	1.2	8.0	0.2	2.6	0.8
		LPDR	0.05	0.04	0.04	0.17	0.01	0.06	0.03
		AE	1.0	0.7	1.1	2.2	−0.9	1.3	0.7
**Smoke mixed with**	**PD**	Alt [km]	4.1	1.4	4.2	8.2	0.9	5.3	3.1
		β [Μm^−1^sr^−1^]	2.3	1.6	1.7	6.6	0.2	3.5	1.0
		LPDR	0.09	0.04	0.08	0.20	0.01	0.11	0.06
		AE	1.1	0.6	1.2	2.5	−0.3	1.5	0.8
	**D**	Alt [km]	2.3	0.5	2.0	3.4	1.7	2.5	2.0
		β [Μm^−1^sr^−1^]	2.9	0.8	2.9	3.9	1.6	3.4	2.0
		LPDR	0.10	0.05	0.09	0.25	0.07	0.13	0.07
		AE	1.2	0.3	1.1	2.0	0.9	1.3	1.0
	**CC**	Alt [km]	5.4	1.5	5.3	9.5	2.2	6.4	5.0
		β [Μm^−1^sr^−1^]	0.7	0.3	0.6	1.8	0.2	0.8	0.5
		LPDR	0.04	0.02	0.04	0.08	0.01	0.04	0.03
		AE	0.8	0.5	0.8	1.5	−0.3	1.1	0.6
	**CP**	Alt [km]	2.8	0.9	2.7	3.9	1.2	3.6	2.3
		β [Μm^−1^sr^−1^]	3.1	1.1	3.0	4.5	1.5	4.2	2.1
		LPDR	0.06	0.04	0.07	0.15	0.01	0.08	0.03
		AE	1.2	0.3	1.2	0.7	1.7	1.4	1.0
	**M**	Alt [km]	N/A	N/A	N/A	N/A	N/A	N/A	N/A
		β [Μm^−1^sr^−1^]	N/A	N/A	N/A	N/A	N/A	N/A	N/A
		LPDR	N/A	N/A	N/A	N/A	N/A	N/A	N/A
		AE	N/A	N/A	N/A	N/A	N/A	N/A	N/A

**Table A2:** Descriptive Statistics for different aerosol types for Region 2 (R2): mean, standard deviation (std), median, maximum value (max), minimum value (min), 75th and 25th percentile for all variables.

Region 2									
	Type	Parameter	Mean	Std	Median	Max	Min	75th perc	25th perc
**Pure**	**S**	Alt [km]	5.1	2.2	5.2	9.6	1.3	6.4	3.3
		β [Μm^−1^sr^−1^]	1.8	1.7	1.0	8.0	0.2	2.8	0.6
		LPDR	0.06	0.04	0.04	0.18	0.01	0.09	0.03
		AE	1.0	0.9	1.1	3.0	−0.9	1.6	0.4
**Smoke mixed with**	**PD**	Alt [km]	5.2	1.1	5.3	7.3	1.8	6.0	4.7
		β [Μm^−1^sr^−1^]	1.2	0.6	1.0	4.0	0.3	1.6	0.8
		LPDR	0.09	0.06	0.09	0.20	0.01	0.12	0.06
		AE	1.4	0.7	1.5	2.8	0.5	1.9	1.1
	**D**	Alt [km]	4.7	0.1	4.7	N/A	N/A	N/A	N/A
		β [Μm^−1^sr^−1^]	3.0	0.2	3.0	3.2	2.9	3.2	2.9
		LPDR	0.18	0.06	0.19	0.23	0.14	0.23	0.14
		AE	1.6	0.1	1.6	1.7	1.6	1.7	1.6
	**CC**	Alt [km]	5.2	1.5	4.6	8.7	3.0	6.0	4.0
		β [Μm^−1^sr^−1^]	1.0	0.7	0.8	2.9	0.4	0.9	0.6
		LPDR	0.05	0.02	0.05	0.10	0.02	0.07	0.03
		AE	0.9	0.6	1.1	1.7	−0.8	1.2	0.8
	**CP**	Alt [km]	1.6	0.6	1.3	2.6	1.2	2.1	1.2
		β [Μm^−1^sr^−1^]	1.6	1.0	1.6	3.4	0.6	2.3	0.7
		LPDR	0.09	0.04	0.11	0.12	0.02	0.12	0.07
		AE	1.1	0.4	1.1	1.6	0.7	1.4	0.7
	**M**	Alt [km]	2.9	0.0	2.9	N/A	N/A	N/A	N/A
		β [Μm^−1^sr^−1^]	1.7	0.0	1.7	N/A	N/A	N/A	N/A
		LPDR	0.05	0.00	0.05	N/A	N/A	N/A	N/A
		AE	0.6	0.0	0.6	N/A	N/A	N/A	N/A

**Table A3:** Descriptive Statistics for different aerosol types for Region 3 (R3): mean, standard deviation (std), median, maximum value (max), minimum value (min), 75th and 25th percentile for all variables.

Region 3									
	Type	Parameter	Mean	Std	Median	Max	Min	75th perc	25th perc
**Pure**	**S**	Alt [km]	5.3	1.5	5.2	9.3	1.8	5.8	4.4
		β [Μm^−1^sr^−1^]	1.1	0.7	0.9	2.9	0.2	1.6	0.6
		LPDR	0.06	0.04	0.05	0.19	0.01	0.79	0.03
		AE	1.1	0.7	1.2	3.2	−0.9	1.6	0.8
**Smoke Mixed With**	**PD**	Alt [km]	4.7	1.2	4.8	7.5	1.3	5.4	3.8
		β [Μm^−1^sr^−1^]	1.5	0.8	0.9	4.2	0.2	1.6	0.6
		LPDR	0.09	0.05	0.05	0.19	0.01	0.08	0.03
		AE	1.4	0.5	1.4	2.7	−0.9	1.6	1.1
	**D**	Alt [km]	4.9	1.6	5.0	7.5	2.0	5.6	3.0
		β [Μm^−1^sr^−1^]	1.8	0.9	2.1	4.5	0.6	2.3	1.0
		LPDR	0.16	0.05	0.08	0.20	0.01	0.11	0.05
		AE	1.4	0.4	1.4	2.3	0.7	1.7	1.0
	**CC**	Alt [km]	4.0	1.9	4.0	8.0	2.5	4.4	2.6
		β [Μm^−1^sr^−1^]	0.9	0.5	1.1	1.6	0.3	1.4	0.4
		LPDR	0.06	0.04	0.04	0.13	0.01	0.09	0.04
		AE	0.9	0.3	0.8	1.4	0.5	1.0	0.7
	**CP**	Alt [km]	1.8	0.6	2.0	2.3	0.7	2.0	1.4
		β [Μm^−1^sr^−1^]	1.3	1.0	0.9	3.3	0.7	0.9	0.8
		LPDR	0.05	0.02	0.05	0.09	0.01	0.07	0.05
		AE	0.7	0.6	0.8	1.2	−0.4	1.0	0.6
	**M**	Alt [km]	3.0	0.3	2.9	3.7	2.8	3.2	2.8
		β [Μm^−1^sr^−1^]	2.6	1.0	2.7	4.1	0.6	3.4	2.3
		LPDR	0.07	0.04	0.06	0.13	0.01	0.12	0.03
		AE	1.5	0.4	1.6	1.9	0.6	1.8	1.3

**Table A4:** Descriptive Statistics for different aerosol types for Region 4 (R4): mean, standard deviation (std), median, maximum value (max), minimum value (min), 75th and 25th percentile for all variables.

Region 4									
	Type	Parameter	Mean	Std	Median	Max	Min	75th perc	25th perc
**Pure**	**S**	Alt [km]	5.5	2.0	5.2	8.8	1.7	6.9	4.3
		β [Μm^−1^sr^−1^]	1.3	0.7	1.2	3.3	0.2	1.7	0.7
		LPDR	0.05	0.03	0.05	0.14	0.01	0.06	0.03
		AE	1.0	0.5	1.0	2.2	−0.1	1.4	0.6
**Smoke Mixed With**	**PD**	Alt [km]	4.8	1.8	4.7	8.2	1.9	6.0	3.2
		β [Μm^−1^sr^−1^]	1.4	0.7	1.3	3.5	0.2	1.6	1.0
		LPDR	0.10	0.05	0.05	0.14	0.01	0.06	0.03
		AE	1.2	0.5	1.1	2.8	−0.4	1.3	1.1
	**D**	Alt [km]	2.4	2.0	N/A	4.7	1.2	3.8	1.2
		β [Μm^−1^sr^−1^]	1.8	0.2	1.8	2.0	1.6	2.0	1.6
		LPDR	0.20	0.04	0.09	0.20	0.02	0.23	0.17
		AE	1.0	1.0	0.5	2.2	0.4	1.8	0.4
	**CC**	Alt [km]	5.6	2.0	4.6	8.0	3.9	8.0	3.9
		β [Μm^−1^sr^−1^]	0.7	0.3	0.5	1.2	0.3	0.7	0.3
		LPDR	0.05	0.04	0.03	0.10	0.01	0.09	0.02
		AE	1.2	0.6	1.1	1.9	0.6	1.7	0.6
	**CP**	Alt [km]	1.1	0.3	1.1	1.4	0.8	1.4	0.8
		β [Μm^−1^sr^−1^]	0.7	0.3	0.7	1.0	0.4	0.9	0.4
		LPDR	0.06	0.06	0.03	0.13	0.01	0.12	0.02
		AE	0.9	0.6	1.1	1.7	0.2	1.3	0.3
	**M**	Alt [km]	N/A	N/A	N/A	N/A	N/A	N/A	N/A
		β [Μm^−1^sr^−1^]	N/A	N/A	N/A	N/A	N/A	N/A	N/A
		LPDR	N/A	N/A	N/A	N/A	N/A	N/A	N/A
		AE	N/A	N/A	N/A	N/A	N/A	N/A	N/A

**Table A5:** Descriptive Statistics: mean, standard deviation (std), median, maximum value (max), minimum value (min), 75th and 25th percentile for all variables regarding the pure smoke layers.

Region	Parameter	Mean	Std	Median	Max	Min	75th perc	25th perc
Region 1	Alt [km]	4.0	1.9	4.0	7.8	1.1	5.8	2.1
	β [Μm^−1^sr^−1^]	2.1	2.0	1.2	8.0	0.2	2.6	0.8
	LPDR	0.05	0.04	0.04	0.17	0.01	0.06	0.03
	AE	1.0	0.7	1.1	2.2	−0.9	1.3	0.7
Region 2	Alt [km]	5.1	2.2	5.2	9.6	1.3	6.4	3.3
	β [Μm^−1^sr^−1^]	1.8	1.7	1.0	8.0	1.0	2.8	0.6
	LPDR	0.06	0.04	0.04	0.18	0.01	0.09	0.03
	AE	1.0	0.9	1.1	3.0	−0.9	1.6	0.4
Region 3	Alt [km]	5.3	1.5	5.2	9.3	1.8	5.8	4.4
	β [Μm^−1^sr^−1^]	1.1	0.7	0.9	2.9	0.2	1.6	0.6
	LPDR	0.06	0.04	0.05	0.19	0.01	0.79	0.03
	AE	1.0	0.7	1.2	3.2	−0.9	1.6	0.8
Region 4	Alt [km]	5.5	2.0	5.2	8.8	1.7	6.9	4.3
	β [Μm^−1^sr^−1^]	1.3	0.7	1.2	3.3	0.2	1.7	0.7
	LPDR	0.05	0.03	0.05	0.14	0.01	0.06	0.03
	AE	1.0	0.5	1.0	2.2	−0.1	1.4	0.6
